# Tarsal coalition resections: a long-term retrospective analysis of 97 resections in 78 patients

**DOI:** 10.1186/s13018-022-03350-8

**Published:** 2022-10-17

**Authors:** Amol Saxena, Ryan Allen, Anthony Wright, Filippo Migliorini, Nicola Maffulli

**Affiliations:** 1 Department of Sports Medicine, Palo Alto Medical Foundation, Palo Alto, CA USA; 2 Seal Beach Podiatry Group, Alamitos, CA USA; 3 Western University, Pomona, CA USA; 4 Department of Orthopaedic, Trauma, and Reconstructive Surgery, RWTH Aachen University Hospital, Pauwelsstr. 30, 52074 Aachen, Germany; 5 Department of Musculoskeletal Disorders, Faculty of Medicine and Surgery, University of Salerno, Salerno, Italy; 6 Queen Mary University of London, Barts and the London School of Medicine and Dentistry, Centre for Sports and Exercise Medicine, Mile End Hospital, 275 Bancroft Road, London, E1 4DG England; 7 School of Pharmacy and Bioengineering, Keele University School of Medicine, Thornburrow Drive, Stoke on Trent, England; 8 Department of Orthopaedic and Trauma Surgery, Eifelklinik St. Brigida, 52152 Simmerath, Germany

**Keywords:** Tarsal coalition, Resection, Pediatric, Talus, Calcaneus, Navicular

## Abstract

**Background:**

Resection of tarsal coalitions provides good patient satisfaction scores, reduced pain, and improved long-term function in both athletic and non-athletic populations. This study aimed to determine when athletic patients undergoing resection of a tarsal coalition were able to return to their desired activity, and whether they experienced a decreased desired activity level (DDA).

**Methods:**

Data on a total of 78 patients who underwent 97 tarsal coalition resections (49 talocalcaneal coalitions, 47 calcaneo-navicular, 14 cuboid-navicular, and three cuneo-navicular; some patients had more than one coalition) operated between January 2001 and June 2020 were prospectively collected. To subjectively assess outcomes, the Roles and Maudsley score (RM) was utilized.

**Results:**

At an average follow-up from the index procedure of 33.6 ± 41.5 months, return to activity for the entire cohort was 18.3 ± 9.6 weeks. Post-RM was 1.3 ± 0.6.

**Conclusion:**

Surgical excision of tarsal coalitions produced favorable results, with most patients able to return to their desired activity level.

**Level of evidence:**

IV.

## Introduction

Tarsal coalitions, first reported in 1769, consist of a fusion between at least two tarsal bones through either osseous, fibrous, or cartilaginous bars [1]. They result from failure of embryonic mesenchyme differentiation and segmentation that follows an autosomal dominant inheritance pattern [1, 2]. Although many different coalitions have been reported, the most common is calcaneo-navicular (CN) coalition, followed by talocalcaneal (TC); together, they account for 90% of all coalitions [1, 3, 4]. A common cause of pain, especially in active patients, with a prevalence of approximately 1–2%, and possibly up to 13%, the condition is bilateral in 50–60% of patients [5–8].

Patients present clinically between 10 and 16 years, with CN coalitions presenting earlier [1, 3, 6], when they start to ossify. Also, at that time, body weight and physical exercise increase [3, 5, 9].

Tarsal coalitions can lead to a valgus hindfoot, flattening of the lateral aspect of the longitudinal arch, muscle spasms, and an overall decrease in motion at the affected joints [1, 3]. These disorders can also cause an increase in foot pain, changes in activity, and an increase in ankle sprains [10]. Correction of other foot deformities should be considered and taken into account with resection [11, 12].

**Fig. 1:**
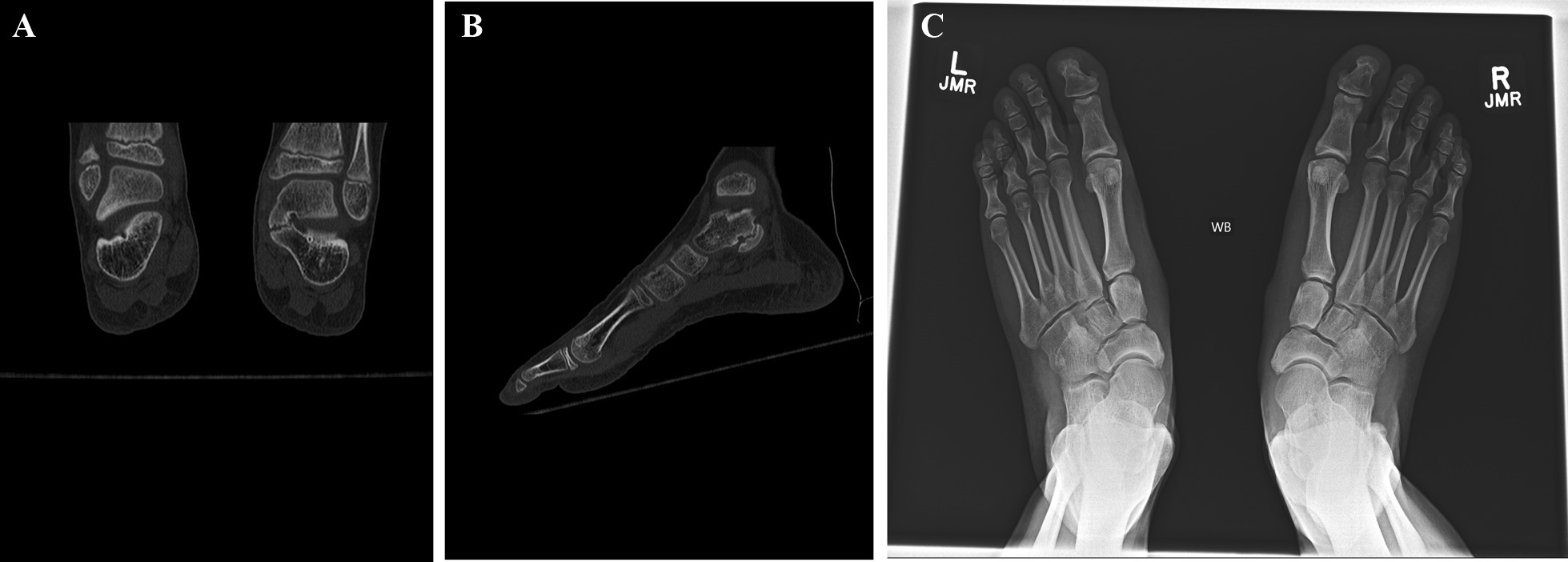
**A** Preoperative radiograph with CN coalition. **B** Preoperative medial oblique plain radiograph with CN coalition. **C** Preoperative lateral plain radiograph with CN coalition

Plain radiography is highly specific for the diagnosis of tarsal coalitions, with 88% and 97% specificity for talocalcaneal and calcaneo-navicular coalitions, respectively [13], (Fig. 1A–C).

**Fig. 2:**
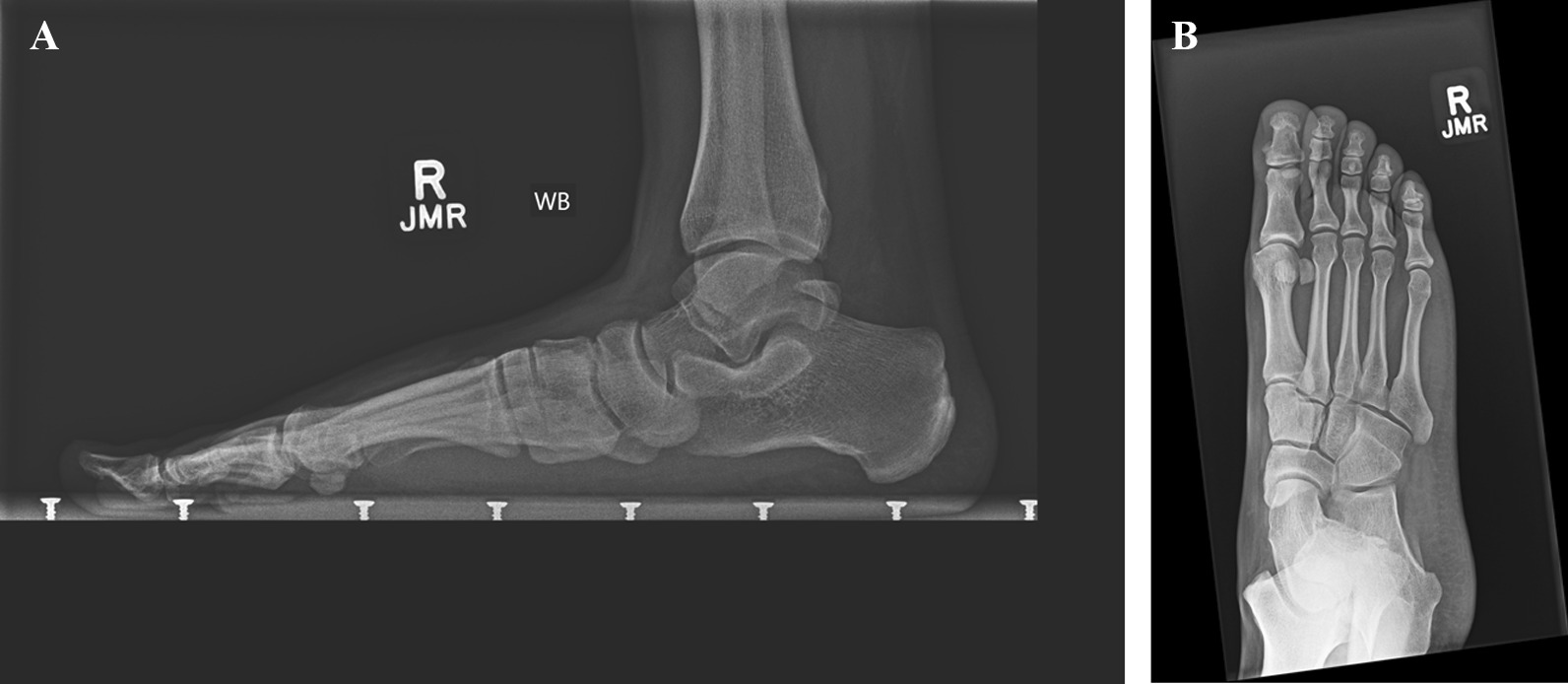
**A** Sagittal view CT of a medial TC coalition. **B** Coronal view CT of bilateral TC coalitions, seen only on the left in this view

Advanced imaging is sought to further evaluate the extent of the coalition, especially when surgery is contemplated. Often, MRI is used, and computed tomography (CT) has been the gold standard to determine the severity of the coalition, demonstrating flattening of the lateral process of the calcaneus, narrowing of the talocalcaneal joint, talar beaking, and narrowing of the sinus tarsi [9, 14], (Fig. 2A, B). CT is useful to evaluate cartilage and bone, while MRI allows to evaluate fibrous or cartilaginous coalitions [15].

Non-surgical management of tarsal coalition includes activity modification, nonsteroidal anti-inflammatories, orthotics, and a period of immobilization in a short leg cast or boot, with surgical intervention following failure of non-surgical treatment [3, 12, 16]. Surgical treatment options include resection or arthrodesis of the affected joint. Resection includes removal of the tarsal coalition, and replacement of the bar with available surrounding structures including fat, tendon or muscle, or bone wax [17–20]. Resection preserves the range of motion without necessarily needing further arthrodesis at a later date [3, 9, 21–23]. In a recent series, although 33.8% of patients needed reoperation following resection, they experienced an average increase of 2.8 points in the visual analogue score (VAS) [24]. Resection of tarsal coalitions in symptomatic athletic patients allowed them to return to the desired activity level, and patients who exhausted conservative measures and decided to forgo surgery are unlikely to reach their desired activity level [9]. Resection produces favorable results when it exceeds 50% of the posterior facet and the hindfoot valgus angle was at least 16 degrees, despite previous studies suggesting arthrodesis in cases of TC bars that exceeded 50% of the total surface area [3, 25]. Arthrodesis, still an accepted alternative, is usually reserved for patients with degenerative joint changes or failed resections [3, 26].

Often concurrent procedures are performed in addition to coalition resections to address other significant deformities; these include subtalar arthroereisis, medial displacement and Evans calcaneal osteotomies, Kidner (posterior tibial tendon advancement) and endoscopic or open gastrocnemius recessions. Subtalar arthroereisis is often used as an isolated procedure to control subtalar joint pronation, often in a flexible pes planovalgus deformity. The calcaneus is in its anatomically correct position when mildly everted (two to four degrees) at maximum pronation for normal and propulsive gait [27]. Therefore, this minimally invasive procedure can be considered for correction of calcaneovalgus and talonavicular fault [28]. Arthroereisis with resection of a talocalcaneal coalition has produced excellent outcomes and is better at correcting the relationship between the talus and calcaneus than resection alone [29]. Heel valgus (frontal plane deformity) can also be treated with a medial displacement calcaneal osteotomy. Forefoot abduction (transverse plane deformity) is treated with Evans calcaneal osteotomy. Medial arch collapse (sagittal plane deformity) is treated with a Kidner procedure. Gastrocnemius equinus is treated with recession techniques. Such procedures in addition to resection of a talocalcaneal coalition showed significant improvement in terms of both VAS and AOFAS scores for the treatment of a rigid pes planovalgus deformity [30].

The present study evaluated the outcome of tarsal coalition resection in terms of time to return to activity and participation in sports. We proposed to use return to activity time frame and an activity-centered patient reported outcome score to assess outcomes, as there is no standardized assessment tools for this condition [1, 9, 10, 12, 31].

## Methods

Data from patients who underwent surgical resection of tarsal coalitions from January 2001 to June 2020 were prospectively collected. Our Institutional Review Board approved the study. All the procedures were performed by (*N* = 89) or directly involved (*N* = 8) the senior author (AS). A total of 97 coalition resection procedures in 78 patients were reviewed by a fellow not involved in the index procedure. Indications for surgical resection were if the patient had rearfoot or ankle pain that either affected the patients’ ability to participate in sports or their daily activities despite non-surgical treatment (insoles, orthoses, braces, and physical therapy). The inclusion criteria documented were return to activity (RTA), age, sex, coalition type resected, additional procedures performed, limb laterality, and complications. To analyze the results, patient satisfaction using the Roles and Maudsley (R&M) scoring system was assessed as it is a patient reported outcome score based on activity level [31]. A four-point scoring system was used to subjectively assess both pain and limitations following a procedure or treatment: 1: no pain or limitations to activity; 2: significant improvement with occasional discomfort but full activity; 3: some improvement but discomfort after prolonged activity; and 4: no improvement from continuous limitation [31]. We also evaluated whether the patients reported decreased desired activity levels (DDA), and whether they returned to their desired activity (RTA), recording the postoperative week when the patients returned to their respective primary sport or activity [9]. Age at the time of surgery and differences between patients below and above 18 were analyzed. Exclusion criteria were patients who did not have data as to when the index procedure was performed, the exact procedure(s), and the location of the coalition.

### Statistical analysis

Student’s t, Fisher’s exact, and Pearson correlation analyses were performed using Excel (Microsoft, Everett, WA, USA) and Stat-Sa (Andover, MA USA) with *P* ≤ 0.05. Confidence interval of 95% was performed when applicable.

### Surgical procedure and postoperative protocol

The procedures were performed with the patient in the supine position for medial talocalcaneal coalitions. The patient was positioned either in the supine position with a bump or laterally with a bean bag for a lateral approach, for example, for a calcaneocuboid coalition. An ankle block with local anesthesia (0.5% bupivacaine) was used in addition to general anesthesia.

For medial talocalcaneal coalitions, the incision was made medially over the subtalar joint, approximately 1.5 to 2 cm long, inferior to the medial malleolus. Care was taken to identify the marginal branches of the greater saphenous vein. The tibialis posterior tendon was retracted dorsally and the flexor digitorum longus tendon was retracted plantarly. After having identified the subtalar joint distally and proximally, an osteotome, rongeur, and small bur were used to resect the coalition, producing a void often of one by one cm, taking care to preserve the articular cartilage. After irrigation, bone wax and gel foam were used in addition to local fat to fill the gap after appreciable increase in motion had been achieved, aiming to reach 25 degrees or more of total subtalar joint range of motion. In later cases, Invuity™ (Stryker, Mahwah, NJ USA) was used to help with visualization.

A modified Ollier incision was used to approach calcaneo-navicular coalitions. Care was taken to identify the intermediate dorsal cutaneous nerve during deep dissection. Dissection was then performed through the extensor digitorum brevis muscle belly, with a portion of the muscle being saved for interposition later. An osteotome, rongeur, and bur were used to excision the coalition. Care was taken to avoid damage to the articular surface of the head of the talus and the calcaneo-navicular ligament. Following irrigation, bone wax, gel foam, and interposed autologous muscle were used to fill the void.

**Fig. 3:**
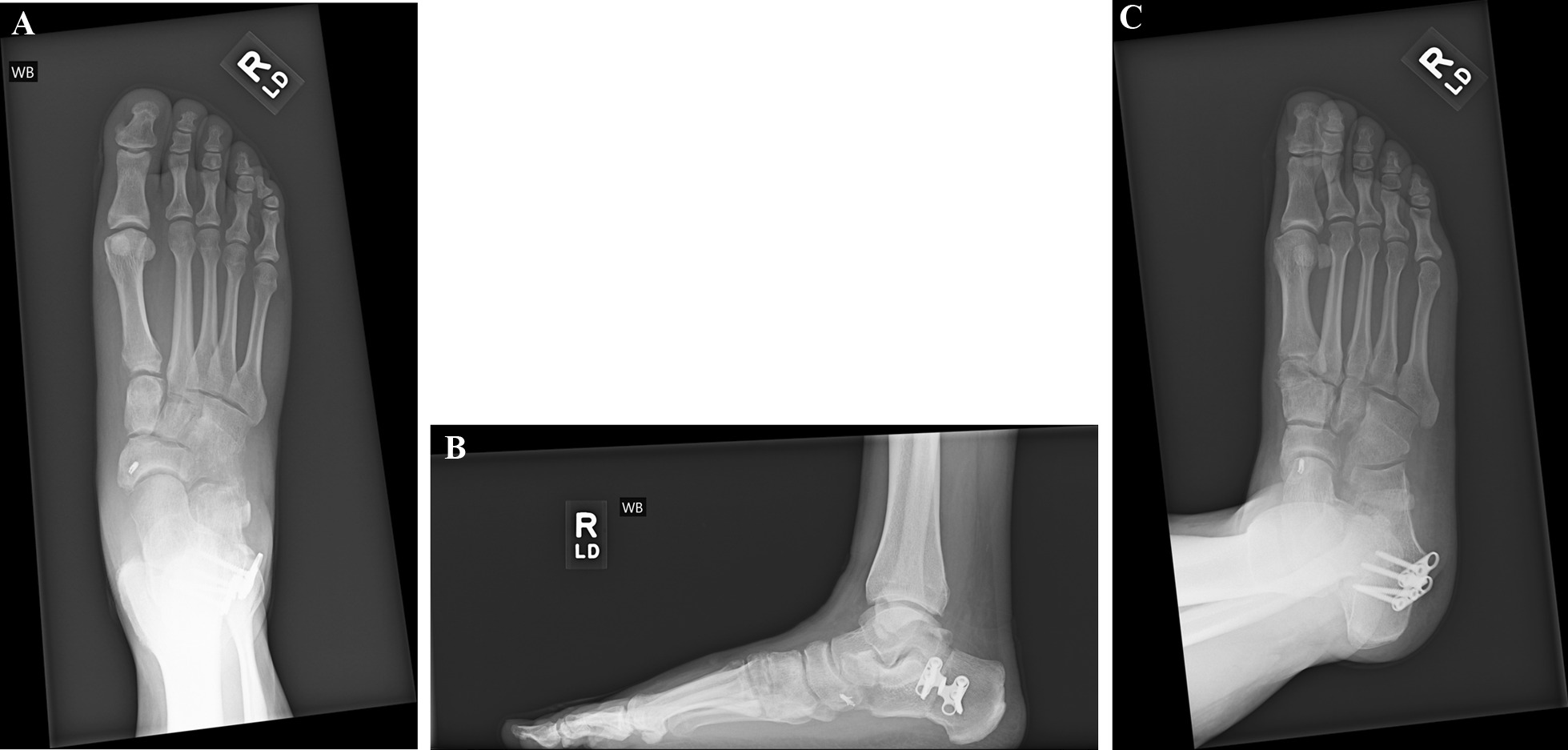
**A** Postoperative plain anteroposterior radiograph; patient is status post-resection of cuboid-navicular coalition, MDP, Evans osteotomy, Cotton osteotomy, Kidner procedure, and an endoscopic gastrocnemius recession. **B** Postoperative plain medial oblique view. **C** Postoperative plain lateral view

Postoperatively, patients were placed in a CAM walker boot. The first follow-up visit was between two and five days. Patients were non-weight bearing for two weeks in either a CAM boot or a well-padded below knee fiberglass cast. Following suture removal, the patient was encouraged to perform range of motion exercises. A CAM boot was then used for two additional weeks in patients who underwent isolated excision of a tarsal coalition. Formal physical therapy was initiated at four weeks postoperatively, with balancing, strengthening, and range of motion exercises. The patients who had undergone a Kidner and MDO procedures would begin weight bearing at four weeks in a CAM boot and then transition to an ankle brace at eight weeks. The patients who had undergone an Evans calcaneal osteotomy started to weight bear at six weeks and transitioned to an ankle brace at 10 weeks. Postoperative films were taken at initial and follow-up visits based on the procedure undertaken (Fig. 3A–C). Physical therapy was initiated at four and six weeks, respectively.

## Results

A total of 97 procedures (48 right and 49 left feet) were performed in 78 patients. Nineteen patients had bilateral procedures, three of them in the same surgical setting. Thirty-four females underwent 41 resections, and 44 males underwent 56 resections. The average age of the entire cohort at the time of surgery was 17.4 ± 10.0 years with an average follow-up from the index procedure of 33.6 ± 41.5 months. The average BMI was 23.5 ± 5.9 (Table 1).

**Table 1:** Entire cohort demographics

Total procedures	97
Total patients	78
*Female	34 (44%)
*Male	44 (56%)
Average Cohort Age (yrs)	17.4 ± 10.0
Average Follow-up (months)	33.6 ± 41.5
Average BMI	23.5 ± 5.9

A total of 49 talocalcaneal, 47 calcaneo-navicular, 14 cuboid-navicular, and three cuneo-navicular coalitions were excised. Fourteen feet had more than one tarsal coalition resected. Eleven patients had a concomitant Evans, and eight had a medial displacement calcaneal (MDO) osteotomies. Twenty-five patients underwent endoscopic gastrocnemius recessions (EGR), and 18 had subtalar arthroeresis (STA).

The procedure that had the closest correlation to both decreased desired activity and degenerative joint disease was the subtalar arthroeresis, followed by the Evans calcaneal osteotomy, medial displacement osteotomy, and endoscopic gastrocnemius recession.

The additional procedures to resection of a tarsal coalitions significantly lengthened the RTA. For the two Kidner procedures, the average age was 24.5 ± 12.02 (range 16–33). The RTA averaged 17.5 ± 9.19 weeks (range 11–24). Both had a post-RM score of one. The age of patients who underwent an endoscopic gastrocnemius recession (*n* = 25) averaged 21 ± 10.89 (range 11–48) years. The RTA averaged 12.71 ± 9.79 weeks (range 8–48). The post-RM score had an average of 1.52 ± 0.90. The age of patients who underwent an Evans procedure (*n* = 11) averaged 21.82 ± 11.90 (range 13–48) years. The RTA had an average of 19.09 ± 7.96 weeks (range 10–35). The RM score had an average of 1.36 ± 0.50. The age of patients who underwent an MDO (*n* = 9) averaged 23.67 ± 12.48 (range 13–48) years. The RTA was 19.89 ± 8.49 weeks (range 10–35). For the post-RM score, the mean was 1.33 ± 0.50. Lastly, for patients receiving an arthroeresis (*n* = 18), the average age was 21.39 ± 11.60 (range 12–48). The RTA was an average of 19.47 ± 10.96 (range 8–48) weeks. The post-RM score had an average of 1.63 ± 1.02. There was no significant difference in RTA and RM based on patients having Evans, MDO, and arthroeresis (Table 2).

**Table 2:** Coalition type and procedural data results

	Average age: (years)	Male	Female	BMI	RTA (weeks)	Post-RM Score
**Coalition type**						
TC (*N* = 49, 52%)	15.7 ± 7.0	30	19	23.1 ± 4.9	18.6 ± 10.1	1.3 ± 0.6
CN (*N* = 46, 48%)	19.5 ± 11.6	33	13	25.0 ± 6.2	14.9 ± 8.2	1.3 ± 0.6
**Associated procedures**						
*Kidner (*N* = 2, 0.1%)	24.5	1	1	26.75	17.5	1
*GR (*N* = 25, 39%)	21	19	6	27.3	12.7	1.5
*Evans (*N* = 11, 17%)	21.8	9	2	25.9	19.1	1.4
*MDCO (*N* = 0, 14%)	23.7	9	2	27.1	19.9	1.3
*Arthroeresis (*N* = 18, 28%)	21.4	15	3	26.9	19.5	1.6

Age at the time of surgery influenced the outcome (Table 3). The average age of patients younger than 18 (*n* = 74) was 13.2 ± 2.3 years, and the average age of patients older than 18 (*n* = 23) was 30.9 ± 13.0 (*P* = 0.00001), with no difference in the duration of postoperative follow-up (*P* = 0.33). The BMI was significantly higher (*P* = 0.0003), as were the levels of DDA (*P* = 0.004) and DJD (*P* = 0.00001) in the older cohort. RTA was not different between the two groups (*P* = 0.67). Post-RM scores were different between the two groups, with the younger cohort scoring better (*P* = 0.005). There were no differences between males and females in terms of duration of postoperative follow-up, age, RTA, and RM scores.

**Table 3:** Differences in return to activity and R&M score by age group

Age	RTA (weeks)	R&M Score
< 18 yo	18.0 ± 10.4	1.2 ± 0.5
>/=18	19.1 ± 6.8	1.7 ± 0.9
*P* value	0.67	0.005

The time to return to activity for the entire cohort was 18.3 ± 9.6 weeks. The post-RM score was 1.3 ± 0.6. All patients’ preoperative RM equaled “4”-pain with daily activities with inability to function. Four patients experienced postoperative DDA (4%), and 12 patients (12%) had postoperative DJD noted on plain films; in 13 patients DJD had been detected pre- or intra-operatively. The 19 patients who had bilateral procedures had an RTA of 20.39 ± 9.55 weeks, whereas the 59 patients who had unilateral procedures returned to activities in 15.7 ± 9.65 weeks (*P* = 0.074), CI 95% (− 0.46 to 9.66).

The population included in the present study was highly active. Sixty-seven of the 78 patients reported to undertake a dedicated sport or activity, with one athlete being a professional runner. The most common sport practiced was soccer, with 21 patients involved. Twenty-one patients were also involved in running; seven belonged to a cross country team, and five to an athletics team. Nine patients were involved in basketball and seven in baseball.

The location of the coalition was not associated with any significant differences in age, RTA, or RM score. The 49 patients in whom a talocalcaneal coalition resection was performed were on average 15.73 ± 7.05 (range 8–45) years old at surgery. The time to RTA averaged 18.58 ± 10.09 (range 6–48) weeks, and the postoperative RM score averaged 1.27 ± 0.57. The patients with 47 calcaneo-navicular coalitions had an average age of 19.46 ± 11.58 (range 8–54) years of age. The time to RTA averaged 14.90 ± 8.18 (range 8–48) weeks. The postoperative RM score averaged 1.33 ± 0.63.

## Discussion

The current study followed a large number of symptomatic tarsal coalitions treated operatively in both pediatric and adult populations. The overwhelming majority of patients were highly active and often involved in team sports. Conservative treatments often fail in the symptomatic athletic population [9]. The outcomes mirror those of other studies which demonstrated good and excellent results, especially in the pediatric age group. Most patients who underwent coalition resections were able to return to playing sports. Four patients in our cohort did not return to their desired activity level. Most patients experienced little to no pain once returning to sports and had few limitations, and the overwhelming majority did not experience a decrease in desired activity level.

This study included the outcomes of adults who underwent resections. While resection is typically performed during adolescence, arthrodesis has historically been the recommended treatment for adults with symptomatic tarsal coalitions [32]. Gonzalez and Kumar reported on 75 calcaneo-navicular coalitions in children [33], with the best results in patients younger than 16 at the time of surgery. Poor results were also seen in those who had degenerative changes of the adjacent talonavicular joint. Patients with cartilaginous coalitions were also noted to experience better outcomes. Cohen et al. evaluated on the success of calcaneo-navicular coalition resections on 13 feet in 12 adult patients [34]. The average age at time of surgery was 33 with the range being 19–48 years. 75% of the patients had degenerative changes. The talonavicular joint was the most frequently involved, followed by cuneo-navicular and subtalar joints. Complications were seen in five of the 13 feet: Three were wound necrosis, and two persistent pain which required an arthrodesis (triple and subtalar). The only treatment failures were in the two patients requiring an arthrodesis. Subjective relief was achieved in all but the two patients who required an arthrodesis.

Our study presents several limitations. Firstly, data were collected prospectively and analyzed retrospectively. Secondly, we utilized the subjective four-point Roles and Maudsley score which does provide a good assessment of activity level. However, we point out that there is no uniformly accepted scoring system to evaluate activity levels after resection of tarsal coalitions. Thirdly, most of our patients were young, with the average age of 17. It is often difficult to achieve a high follow-up rate on patients in this age group, considering the issue of relocation after high school for employment or higher education. Nonetheless, we were able to follow up our cohort for an average of 33 months. Another strength was the size of the patient population (*N* = 78) and the number of resections (*N* = 97). Unlike most other studies which analyzed the resection of tarsal coalitions alone, the current study also reviewed common concurrent procedures to address pes planovalgus deformities, including subtalar arthroeresis, endoscopic gastrocnemius recessions, and calcaneal osteotomies, performed when tarsal coalition resection is performed.

## Conclusion

In conclusion, our study demonstrated that resections of tarsal coalitions had a favorable outcome for the overwhelming majority of patients. In the present study, return to activity was approximately 18 weeks, and this value included patients who underwent concurrent procedures such as a calcaneal osteotomy. Patients in whom a talocalcaneal coalition was excised returned to activity on average 18.6 weeks after the index procedure, and those in whom a calcaneo-navicular coalition was excised returned to activity sooner, at 14.9 weeks. These values should provide information in terms of ability to return to activity and time frame of recovery and can also help with surgical timing to, for example, plan surgery between sports seasons, as many of the patients were involved in several sports.

## Data Availability

This study does not contain any third material.

## Abbreviations

DDA: Decreased desired activity
RM: Roles and Maudsley score
CN: Calcaneo-navicular
TC: Talocalcaneal
VAS: Visual analogue scale
CT: Computed tomography
